# What the Local Centres Are Doing

**Published:** 1921-01-22

**Authors:** 


### THE COLLEGE OF NURSING

*(Limited by Guarantee.)*

### WHAT THE LOCAL CENTRES ARE DOING.

### LONDON CENTRE.

(Secretary, Miss Bompas, 7 Henrietta Street, W. 1.)

Forthcoming Elections.

Members are reminded of the important general meeting of the London Centre, of which we gave particulars last week, to be held on Tuesday, January 25, at 8 p.m., at Guy's Hospital. The subject for discussion is the policy of the Centre with reference to the forthcoming election of Council members. London Centre members wishing to discuss the coming election of Council members are asked to come to the Club Room on Friday, January 21, at 5.30 p.m., for an informal talk on the subject.

Educational Classes.

The fee for French Classes for a course of three months is £1 10s., and for the Voice Training and Speaker's Class, held by Miss Umfreville Hicks, the fee for the course of ten lessons is £5 5s. for a class of ten members. Full particulars of these classes were given in our columns last week.

### CAMBRIDGE CENTRE.

(Hon. Secretary, Miss E. A. Lennard, Thornleigh, Hill's Avenue.)

THE NEXT LECTURE.

Friday, February 11, 3.15 p.m., at Addenbrooke's Hospital, lecture on "The Treatment of Hysteria," by Dr. Archdale. It is hoped there will be a large attendance.

A Conducted Museum Visit.

On Wednesday, January 12, some of the members of this Centre visited the Pathological, Surgical, and Medical Museum, by kind permission of Professor Woodhead, and were taken round by the Assistant Curator, who spared no pains in showing all the interesting and wonderful specimens. It was a most informative afternoon, and it was unfortunate that only a small number of members could be present.

### LEICESTER CENTRE.

(Hon. Secretary, Miss Masters, Poor-Law Infirmary, Gwendolen Road, Leicester.)

Forthcoming Lectures.

Tuesday, January 25, at 6 p.m., T. C. Clare, Esq., M.D., F.R.C.S., "Gynæcological Nursing."

Tuesday, February 8, at 6 p.m., Miss M. H. Cowlin, Director of Women Police Patrols, Liverpool, "Police Work as a Profession for Women."

Tuesday, February 22, at 6 p.m., Miss Clephan, "Internationalism and the League of Nations."

Tuesday, March 8, at 6 p.m., W. W. Mackarell, Esq., M.D., Ch.B., D.P.H., D.P.M., on "Vaccines and Serums."

Tuesday, March 22, at 6 p.m., Colonel C. J. Bond, C.M.G., F.R.C.S., on "The Consultative Council's Scheme for an Improved Medical Service in Relation to Nursing."

Season ticket for the course: Members, 2s. 6d.; non-members, 4s.

### SWANSEA AND SOUTH WALES CENTRE.

(Hon. Secretary, Mrs. F. W. Jenner, Glynn Vivian Home of Rest for the Blind, Caswell Hill, Mumbles.)

Sir Arthur Stanley to Speak.

On Friday, January 28, in the Y.M.C.A., Swansea, Doctor Mary Scharlieb, C.B.E., will speak on "Venereal Disease as it Affects Women and Children."

On the following day, Saturday, January 29, at 3 p.m., Sir Arthur Stanley, G.B.E., C.B., will speak in the Y.M.C.A. on "The Formation of the College of Nursing, its Aims and Objects." Sir Arthur Stanley will be accompanied by Miss Sheriff Macgregor, Organising Secretary of the College. Members of the Centre from outlying districts who would like hospitality for the night of January 28 should write at once to the Secretary.

### SHEFFIELD CENTRE.

(Hon. Secretary, Mrs. Barnes, 34 Wilkinson Street, Sheffield.)

With a view to augmenting the funds for the establishment of a Nurses' Club in the centre of the city, a Ball is to be held on Monday, January 24. Mrs. Watson, Hon. Treasurer of the local Centre, hopes there will be a good attendance. Tickets may be obtained at all the local hospitals.

### THE COLLEGE IN IRELAND.

A CENTRE FOR BELFAST.

It was resolved at a largely attended public meeting, held on January 5, at the Medical Institute, Belfast, to form a Local Centre of the College of Nursing, to be called the Ulster Centre. Professor R. J. Johnstone, F.R.C.S., who presided, pointed out that the new status conferred on nurses by the Registration Acts imposed on them certain duties, and Dr. J. B. Moore, in moving the establishment of the new Centre, said that he felt it would be an extremely useful thing to the nurses of Belfast and surrounding district to have a Local Centre there; while Sir Andrew Horne, in supporting the resolution, remarked that it was probable that a Parliament would be established in Belfast, and there was no reason why, when the College organisation was complete, some of its members should not become active members of that Parliament, have a voice in its affairs, and look after the interests of nursing throughout the whole country. Miss Sheriff Macgregor, R.R.C., the Organising Secretary of the College, spoke of the importance of character in a nurse, and following on that, of the importance of education. Nursing should be so organised as to attract women of character and good education, and the College had proved of great value to the nurse in helping her to educational advantages by granting studentships and scholarships. Nursing ideals should be not only moral and intellectual, but they should include economical protection for the nurse, who should be paid at such a rate as to allow her to be a self-respecting citizen. Nothing, however, could be done without effort, and nurses should support the College and its Centres in the College's effort to realise its ideals for the profession.

The Countess of Clanwilliam was elected President of the Ulster Centre, Miss Gray Hon. Treasurer, Dr. John Macintosh, O.B.E., Hon. Secretary, and the following to be members of the Executive Committee: The Misses McClure, Slavin, Jelly, Douglas, Knox, Burnside, Snodgrass, Woods, Seaton, Higginson, Gault, Fulton, and Queen, together with the Ulster members of the Irish Board.

THE IRISH BOARD.

A special meeting of the Irish Board of the College of Nursing was held at the offices, 54 Fitzwilliam Square, Dublin, on January 13, at which Sir Andrew Horne presided. The resolution passed by the Executive and Finance Committee on January 4, expressing sorrow and deep regret at the lamentable death of the Chairman of the Board, Dr. George Peacocke, was confirmed. Dr. Peacocke had done much to advance the interests of the College in Ireland, and his death, the resolution states, will be a grave and almost irreparable loss to the Irish Board.

Sir Andrew Horne, Vice-Chairman of the Irish Board since its foundation, was unanimously elected to be Chairman in succession to the late Dr. Peacocke, and Miss Hill, Matron of the Adelaide Hospital, Vice-Chairman, in succession to Sir Andrew Horne. Miss Philan, Lady Superintendent, Dublin Union, was elected a member of the Board.

In future the Board's meetings will be held monthly instead of quarterly.

